# Lunasin Sensitivity in Non-Small Cell Lung Cancer Cells Is Linked to Suppression of Integrin Signaling and Changes in Histone Acetylation

**DOI:** 10.3390/ijms151223705

**Published:** 2014-12-18

**Authors:** Junichi Inaba, Elizabeth J. McConnell, Keith R. Davis

**Affiliations:** 1 Owensboro Cancer Research Program, Mitchell Memorial Cancer Center, Owensboro, KY 42303, USA; E-Mails: jinaba@ocrp.org (J.I.); emcconne5561@gmail.com (E.J.M.); 2 James Graham Brown Cancer Center and Department of Pharmacology & Toxicology, University of Louisville, Louisville, KY 40202, USA

**Keywords:** lunasin, lung cancer, integrin signaling, histone acetylation, cell proliferation, Akt signaling

## Abstract

Lunasin is a plant derived bioactive peptide with both cancer chemopreventive and therapeutic activity. We recently showed lunasin inhibits non-small cell lung cancer (NSCLC) cell proliferation in a cell-line-specific manner. We now compared the effects of lunasin treatment of lunasin-sensitive (H661) and lunasin-insensitive (H1299) NSCLC cells with respect to lunasin uptake, histone acetylation and integrin signaling. Both cell lines exhibited changes in histone acetylation, with H661 cells showing a unique increase in H4K16 acetylation. Proximity ligation assays demonstrated lunasin interacted with integrins containing αv, α5, β1 and β3 subunits to a larger extent in the H661 compared to H1299 cells. Moreover, lunasin specifically disrupted the interaction of β1 and β3 subunits with the downstream signaling components phosphorylated Focal Adhesion Kinase (pFAK), Kindlin and Intergrin Linked Kinase in H661 cells. Immunoblot analyses demonstrated lunasin treatment of H661 resulted in reduced levels of pFAK, phosphorylated Akt and phosphorylated ERK1/2 whereas no changes were observed in H1299 cells. Silencing of αv expression in H661 cells confirmed signaling through integrins containing αv is essential for proliferation. Moreover, lunasin was unable to further inhibit proliferation in αv-silenced H661 cells. This indicates antagonism of integrin signaling via αv-containing integrins is an important component of lunasin’s mechanism of action.

## 1. Introduction

Lunasin, a peptide present in crude soy protein, has been proposed to be an important chemoprevention agent in soy [1,2,3]. Lunasin is a 43-44 amino-acid peptide encoded within the soybean GM2S-1 gene [4,5]. It contains a 22 amino acid *N*-terminal sequence with no known function followed by a putative helix domain proposed to target lunasin to chromatin, and a *C*-terminal end that includes a RGD cell-adhesion motif followed by a poly-aspartic acid tail [4,6]. Lunasin’s potential chemoprevention activity was established by studies showing lunasin prevented cellular transformation by chemical carcinogens and viral oncogenes [6,7,8,9]. More recent studies have shown lunasin can inhibit the growth of breast [10,11], leukemia [12], colon [13] and lung cancer [14] cells *in vitro* and *in vivo*. Taken together, these results suggest lunasin may have the potential to be used as a cancer therapeutic agent.

The mechanism of action (MOA) responsible for lunasin’s anticancer activity is currently not clearly defined. Once lunasin is internalized [15], it enters the nucleus and binds to hypoacetylated regions of chromosomes such as the telomeres [1,9]. Lunasin binds to the deacetylated core histones H3 and H4 *in vitro* and current hypotheses on lunasin’s MOA suggest this is critical for the anticancer effects of lunasin [7,8,16,17,18,19]. Current models of lunasin’s MOA focus on the disruption of normal histone acetylation and a concomitant disruption of cell cycle regulation or induction of apoptosis [20,21]. Lunasin-induced apoptosis in cancer cells may be through the intrinsic pathway [12,13] and involve the tumor suppressor phosphatase and tensin homolog (PTEN) [22]. Lunasin also has anti-inflammatory activity that may be mediated by suppression of the nuclear factor kappa-light-chain-enhancer of activated B cells (NF-κB) pathway [23,24]. Gene expression studies indicate lunasin affects a number of signaling pathways in different cell types, thus, some of the observed biological effects of lunasin may be independent of histone acetylation [21,25].

Since lunasin contains a RGD domain, it has been suggested in some cell types, lunasin may bind to integrins that recognize this cell adhesion motif [1,15,26,27]. Integrins are heterodimeric cell-surface proteins that play critical roles in adhesion to the extracellular matrix and transmitting extracellular signals that affect cell migration and the regulation of signaling pathways involved in cell survival and proliferation. Although these studies on lunasin’s interaction with integrin pathways and modulation of histone acetylation provide important clues into the potential mechanisms whereby lunasin influences cell proliferation and viability, the current models are highly speculative and functional studies are required to clearly delineate lunasin’s MOA. We have recently shown that lunasin has cell-specific effects on the proliferation of non-small cell lung cancer (NSCLC) cells and that NSCLC line H661 is sensitive to lunasin whereas H1299 is resistant when cultured under adherent culture conditions [14]. The inhibition of proliferation H661 cells by lunasin was found to be due to a block at the G1/S phase that was caused by disruption of regulatory phosphorylations of the retinoblastoma protein. Here, we demonstrate lunasin’s ability to block the G1/S phase transition in non-small cell NSCLC H661 cells is due at least in part to its ability to bind specific integrins and inhibit integrin signaling pathways.

## 2. Results

### 2.1. Lunasin Sensitivity Is Associated with Increased Lunasin Uptake

**Figure 1:**
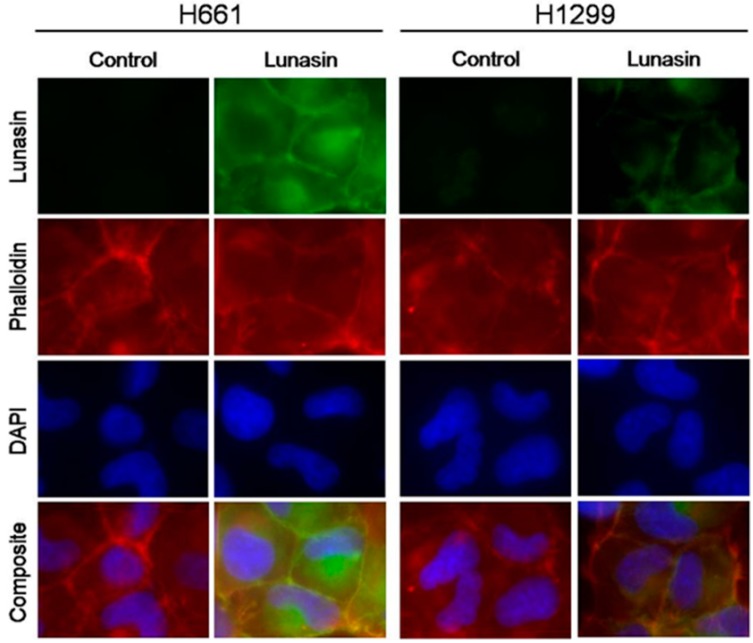
Internalization of lunasin into non-small cell lung cancer (NSCLC) cells. Cells were treated for 24 h with either vehicle (Control) or 100 µM lunasin prior to processing for immunocytochemistry.

Given that one potential mechanism for lunasin effects on cells is based on the interaction of lunasin with histones and modulating of histone acetylation, we performed detailed immunocytochemistry studies comparing the internalization of lunasin in lunasin-sensitive H661 and lunasin-insensitive H1299 cells. These studies utilized our mouse monoclonal anti-lunasin antibody, a fluorescently-labelled phalloidin probe to visualize actin, and 4',6-diamidino-2-phenylindole (DAPI) staining to identify nuclear regions. These analyses clearly show lunasin is internalized in both H661 and H1299 cells; however, significantly higher levels of lunasin were detected in H661 cells (Figure 1). Interestingly, a significant amount of the lunasin detected was located in the cytoplasm at 24 h. Thus, lunasin sensitivity is correlated with significantly higher levels of internalized lunasin.

### 2.2. Lunasin Binds Histones in Situ and Affects Histone Acetylation

**Figure 2:**
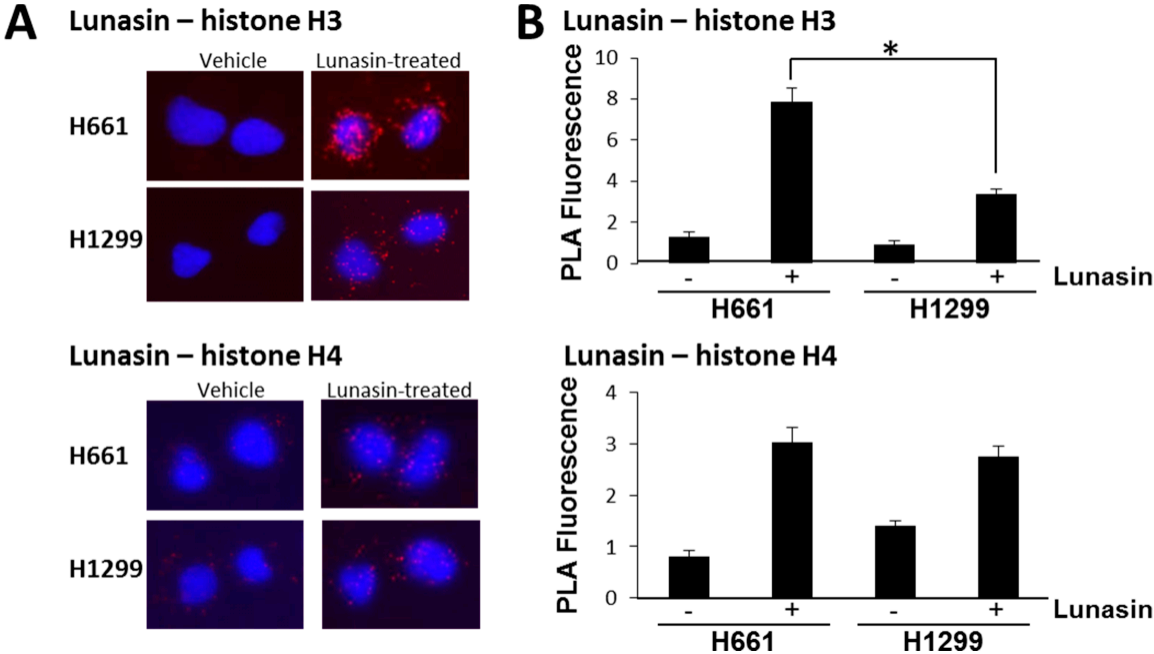
(**A**) Interaction of lunasin with core histones H3 and H4 in NSCLC cells. Cells were treated with 100 µM lunasin for 24 h before performing proximity ligation assays (PLA) assays using antibodies specific for lunasin, H3 and H4; (**B**) Quantitation of PLA fluorescence in NSCLC cells. Fluorescence is expressed as relative fluorescence per cell. Data shown are the mean ± SD obtained in three independent experiments where 40 cells per treatment were imaged in each experiment; asterisks indicate a statistically significant difference (*p* < 0.05) between treatments.

To determine if lunasin binds core histones in NSCLC cells and affects histone acetylation, we compared the response of the most sensitive line, H661, to a lunasin-resistant line, H1299. Cells were treated with 100 µM lunasin for 24 h and the binding of lunasin to histones H3 and H4 *in situ* was measured via proximity ligation assays (PLA, [28]). This concentration of lunasin has previously been shown to inhibit H661 proliferation by approximately 50% [14]. PLA assays demonstrated lunasin does indeed interact with both histones H3 and H4 *in vivo* (Figure 2). Lunasin-histone interactions were detected in both H661 and H1299 cells, with the amount of interaction with H3 being significantly higher in H661 cells. Interestingly, the amount of interaction of lunasin with H4 was lower than H3 and was similar in both H661 and H1299 cells. These results confirm and extend previous studies that lunasin interacts with core histones and demonstrates the amount of interaction with H3 is higher in lunasin-sensitive cells compared to lunasin-resistant NSCLC cells.

**Figure 3:**
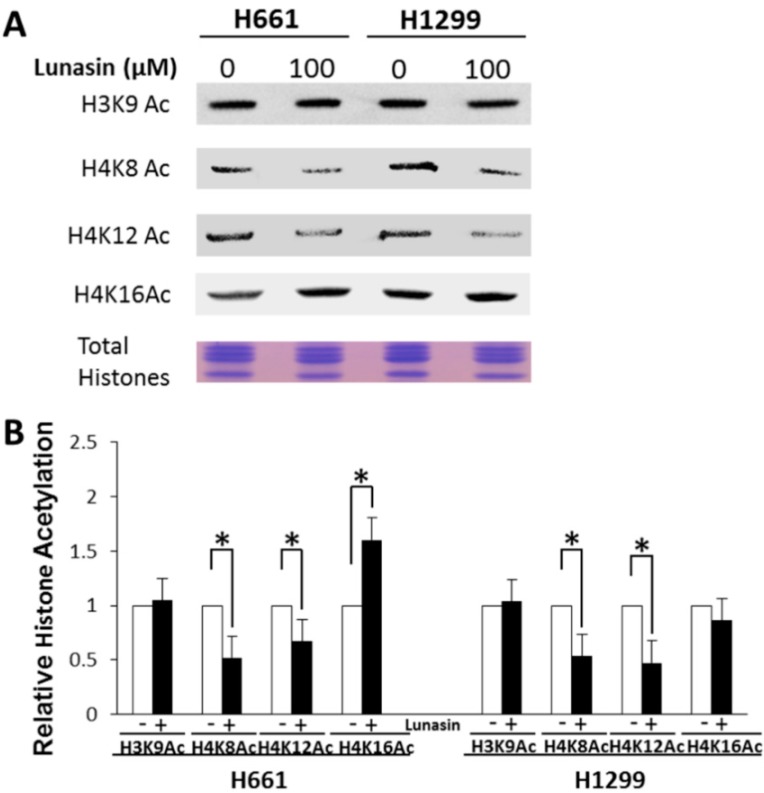
(**A**) Immunoblot analysis of acetylated histones in lunasin-treated and untreated NSCLC cells; (**B**) Relative histone acetylation in lunasin-treated and untreated NSCLC cells. Cells were treated with 100 μM lunasin or vehicle for 48 h. Total histones were isolated and subjected to immunoblot analyses using antibodies specific for the indicated histone acetylation marks. Relative histone acetylation was determined by image analyses of immunoblots using Image J software. Data shown are the mean ± SD of immunoblots obtained in three independent experiments; asterisks indicate a statistically significant difference (*p* < 0.05) between treatments.

Histone acetylation was then evaluated by isolating total histones and performing immunoblot analysis using antibodies specific for histone acetylation marks known to be important for modulating gene expression. Lunasin-treated H661 and H1299 cells exhibited significant changes in histone acetylation compared to buffer-treated controls (Figure 3). Both H661 and H1299 exhibited a similar significant decrease in acetylation at H4K8 and H4K12c compared to controls and neither cell line showed a significant change in H3K9 acetylation after lunasin treatment. However, the acetylation of H4K16 was significantly higher in lunasin-treated H661 cells, whereas acetylation at this mark was not affected in H1299 cells. Thus, lunasin treatment does affect the acetylation status of both lunasin-sensitive and insensitive NSCLC cells and lunasin sensitivity is correlated with increased acetylation at H4K16.

### 2.3. Interaction of Lunasin with Specific Integrin Subunits

**Figure 4:**
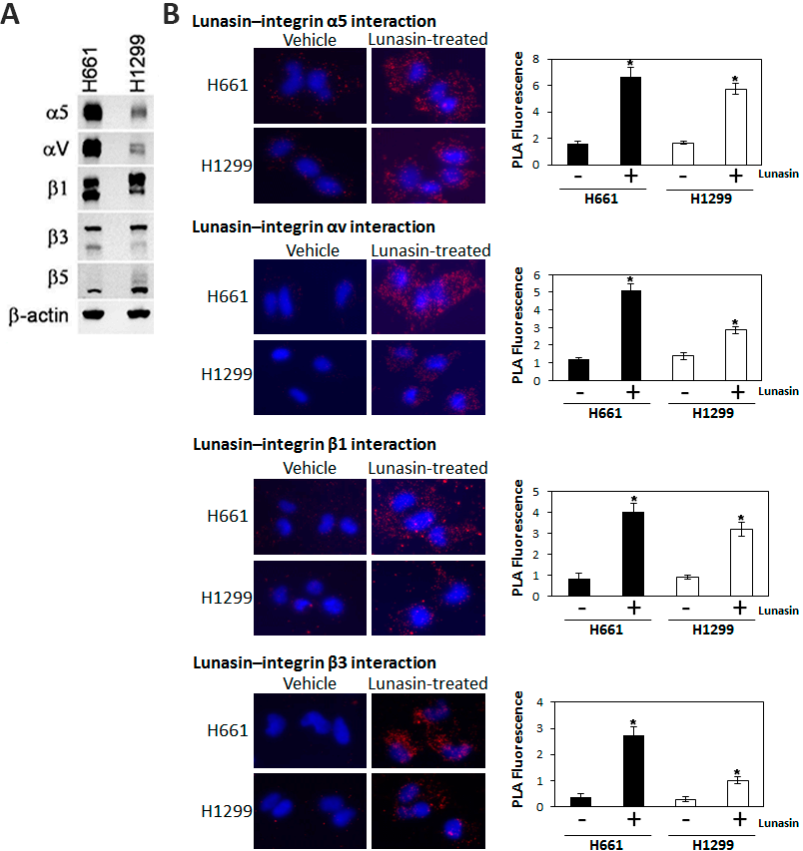
(**A**) Analysis of integrin subunit expression in NSCLC cell lines H661 and H1299. Protein extracts were subjected to immunoblot analysis using antibodies specific for the indicated integrin subunits; (**B**) Detection of lunasin interactions with specific integrin subunits *in situ*. **Left**-hand panels show representative PLA analyses of lunasin and the indicated integrin subunit; **Right**-hand panels show the quantitation of fluorescence for each interaction. Fluorescence is expressed as relative fluorescence per cell. Data shown are the mean ± SD obtained in three independent experiments; asterisks indicate statistically significant difference (*p* < 0.05) from the vehicle-treated control.

Since lunasin contains an RGD domain and earlier studies suggested lunasin may bind integrins, we used immunoblotting analyses to measure the levels of specific integrin subunits in H661 and H1299 cells to see if there was a significant difference in their integrin expression profiles. Both cell lines expressed all of the integrin subunits that were probed; however, there were significant differences in the relative levels expressed in each cell line (Figure 4A). H661 expressed higher levels of αv and α5 compared to H1299, with the αv and α5 subunits being dramatically lower in H1299 cells. The β1 and β3 subunits were expressed at similar levels in both cell lines, with β1expression being higher than β3. The β5 subunit was expressed at a relatively low level in both cell lines, but was more highly expressed in H1299 cells. Based on these results, we performed a series of PLA studies to determine if lunasin interacts with specific integrin subunits *in vivo*. Cells were treated with 100 µM lunasin for 24 h and subjected to PLA using antibodies specific for lunasin and the integrin subunits αv, α5, β1 and β3. PLA signals that were significantly higher than the background levels observed in vehicle treated cells were observed for all four integrin subunits tested (Figure 4). The extent of lunasin interactions with α5 and β1were similar in both H661 and H1299 whereas the extent of lunasin interactions with αv and β3 were higher in H661 compared to H1299. Thus, lunasin sensitivity correlated with increased interactions with αv and β3.

### 2.4. Lunasin Disrupts Integrin Signaling

**Figure 5:**
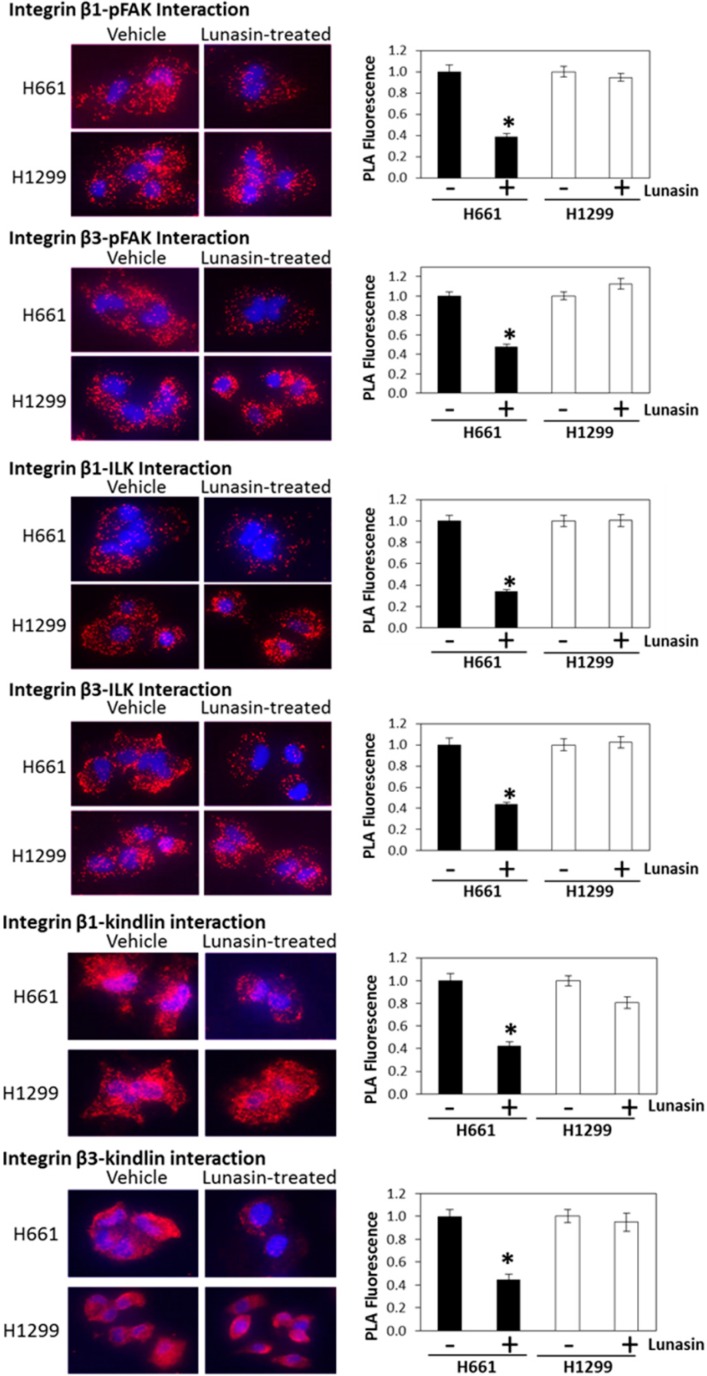
Detection of integrin β subunit interactions pFAK, Kindlin and ILK. Left-hand panels show representative PLA analyses of lunasin and the indicated integrin subunit. Right-hand panels show the quantitation of fluorescence for each interaction. Fluorescence is expressed as relative fluorescence per cell. Data shown are the mean ± SD obtained in three independent experiments; asterisks indicate statistically significant difference (*p* < 0.05) from the vehicle-treated control.

Since lunasin was found to interact with specific integrin subunits, we used two approaches to investigate whether lunasin had any effects on integrin signaling. We first used PLA to determine if lunasin treatment affected the interaction of integrin β subunits with the downstream signaling effectorspFAK, Integrin Linked Kinase (ILK) and Kindlin. These studies clearly demonstrate the interaction of integrins containing either β1 or β3 with all three signaling partners were significantly reduced in H661 cells, but not in H1299 cells (Figure 5). Based on fluorescence measurements, the interaction of both β1 and β3 with pFAK, ILK, and Kindlin was inhibited by 50%–70% in H661 cells by lunasin treatment.

**Figure 6:**
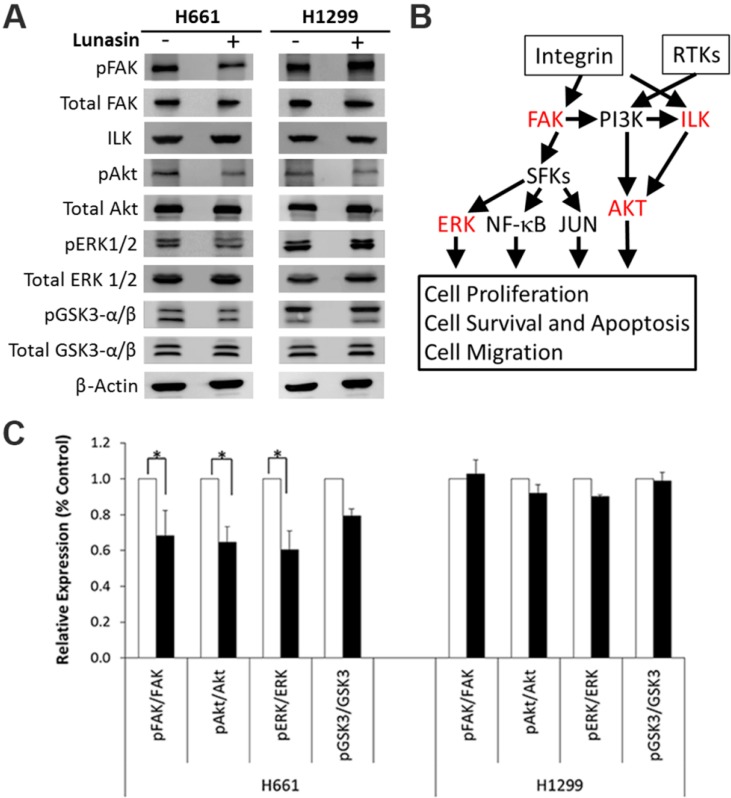
(**A**) Immunoblot analyses of integrin signaling pathway components in lunasin-treated NSCLC cells. Analyses were repeated in three independent experiments and representative data from one experiment is shown; (**B**) Integrin signaling pathway showing effects of lunasin treatment in H661 cells. RTKs, Receptor Tyrosine Kinases; SFKs, Src Family Kinases. Proteins shown in red indicate signaling steps negatively affected specifically in H661 cells by lunasin treatment; (**C**) Relative expression levels of integrin signaling proteins. Immunoblots shown in (**A**) were analyzed using ImageJ software v1.45 (National Institutes of Health, Bethesda, MD, USA). Data represent the mean ± SD for three independent experiments. The asterisk (*) indicates a significant (*p* < 0.05) difference in expression levels relative to the vehicle control.

To confirm lunasin disrupted integrin signaling in H661 cells, we performed immunoblot analyses to measure the levels of several downstream signaling components including FAK, ILK, Akt, ERK1/2 and GSK3-α/β. Lunasin treatment of the lunasin-insensitive H1299 cells did not have a significant effect on the steady-state levels of any of these signaling components, nor were there any effects on the phosphorylation of FAK, Akt, ERK1/2 or GSK3-α/β. Similarly, lunasin treatment of the lunasin-sensitive H661 cells did not significantly affect steady-state levels of any of these signaling components; however, reductions in FAK, Akt, and ERK1/2 phosphorylation were reproducibly observed (Figure 6). These results confirm lunasin does suppress integrin signaling in lunasin-sensitive H661 cells.

### 2.5. The Alpha-v Integrin Subunit Is Required for NSCLC H661 Cell Proliferation

**Figure 7:**
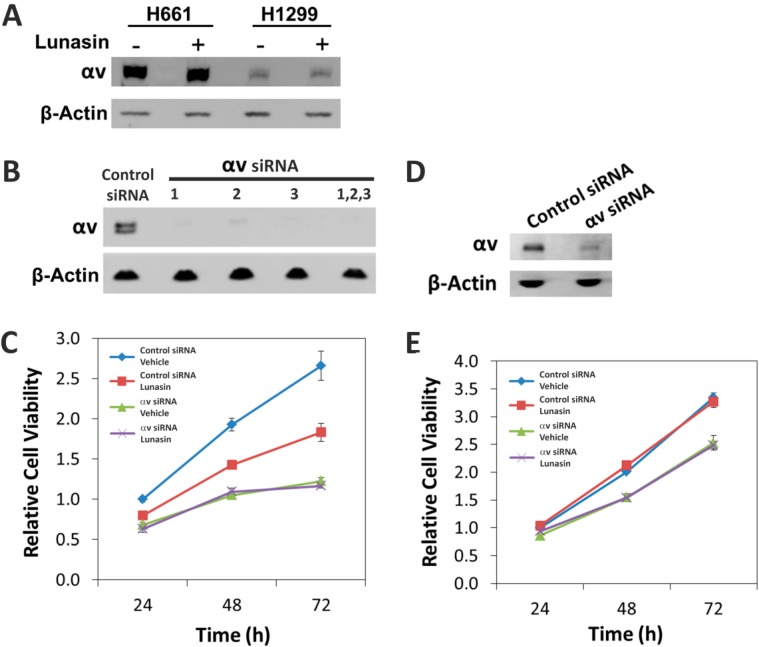
Effects of silencing the αv integrin subunit in NSCLC H661and H1299 cells. (**A**) Immunoblot analyses of αv subunit levels in H661 and H1299 cells treated with either vehicle or lunasin. Cells were treated for 24 h with 100 µM lunasin or 50 mM NaPO_4_, pH 7.4; (**B**) Immunoblot analysis of αv expression in H661 cells transfected with a control siRNA or three different siRNAs designed to silence αv expression individually or in combination. β-actin was used as a loading control; (**C**) Proliferation of control siRNA transfected H661 cells and H661 cells transfected with a combination of three αv-specific siRNAs; (**D**) Immunoblot analysis of αv expression in H1299 cells transfected with a combination of three αv-specific siRNAs; (**E**) Proliferation of control siRNA transfected H1299 cells and H1299 cells transfected with a combination of three αv-specific siRNAs. Proliferation assays were initiated 48 h after transfection with siRNAs; cells were treated with either 50 mM NaPO_4_, pH 7.4 (vehicle) or 100 µM lunasin. Data shown are the mean ± SD obtained in three independent experiments.

Since our PLA studies demonstrated lunasin sensitivity was correlated with higher levels of lunasin interaction with the αv integrin subunit, we hypothesized that lunasin’s ability to inhibit proliferation in H661 cells was due at least in part, on disruption of signaling through αv-containing integrins such αvβ3. We first tested whether lunasin treatment altered the expression of the αv subunit. Immunoblot analysis confirmed H1299 cells does indeed express significantly lower levels of αv protein (Figure 7A), which is consistent with the previous immunoblot studies (Figure 4A) and PLA assays (Figure 4B) that detected lower amounts of lunasin-αv interaction. To functionally test whether the αv integrin subunit was required for lunasin action, we utilized siRNAs to silence αv expression in H661 and H1299 cells. Three different siRNA constructs were found to efficiently silence αv expression at the protein level in H661 cells, and the combination of all three siRNAs reduced αv protein accumulation to undetectable levels in H661 cells (Figure 7B) and by approximately 75% in H1299 cells (Figure 7D). To determine the effects of αv silencing on the ability of lunasin to inhibit proliferation of H661 and H1299 cells, the growth of control siRNA-treated cells with αv-silenced cells were compared with and without lunasin treatment. As previously observed [14], lunasin treatment of cells transfected with the control siRNA caused a significant reduction in proliferation of H661 cells over a 72 h treatment period (Figure 7C, Supplementary Figure S1) whereas lunasin did not affect proliferation of H1299 cells. H661 cells transfected with αv siRNAs showed a significant reduction in proliferation that was not further increased by lunasin treatment. H1299 cells transfected with αv siRNAs showed a 30% reduction in proliferation that also was not further increased by lunasin treatment. These results clearly demonstrate the αv integrin subunit is required for maximum proliferation of H661 cells and that lunasin cannot further inhibit proliferation in the absence of αv-containing integrins. Proliferation of H1299 cells appears less dependent on αv expression and modifying the amount of αv present does not change the lunasin-insensitive phenotype of this cell line.

## 3. Discussion

A growing body of evidence strongly suggests lunasin has the ability to inhibit the growth of several diverse cancers [3,29] and our recent studies have extended lunasin’s potential use to treating NSCLC [14]. We have now taken advantage of the differential lunasin sensitivity of two NSCLC cell lines in adherent culture to investigate several potential mechanisms that may be important for mediating lunasin’s effects. Immunofluorescence localization studies demonstrated lunasin accumulated to higher levels in the lunasin sensitive H661 cells compared to the lunasin-insensitive H1299 cells at 24 h after lunasin treatment. Most of the lunasin accumulated in the cytoplasm of both cell lines at this treatment time, which is consistent with previous studies in colon cancer cells [27] and the rapid uptake of lunasin in macrophages [15]. The initial accumulation in the cytoplasm is likely related to lunasin uptake being mediated via integrin binding and endocytosis through clathrin-coated vesicles [15]. One interesting and important unanswered question is if and how lunasin is released from the endocytic pathway so that it might interfere with histone acetylation [16,19,21].

Given earlier reports supporting effects of lunasin on histone acetylation as being a primary mechanism for lunasin action, we investigated whether the differential sensitivity of the NSCLC cell lines was related to histone binding and histone acetylation. PLA studies demonstrated the amount of lunasin-H3 interaction was significantly higher in the lunasin-sensitive H661 cells compared to H1299. In contrast, the amount of lunasin-H4 interaction was similar in both cell lines. Strikingly, the observation that lunasin-histone interactions were clearly localized in the cytoplasm opens up the possibility that lunasin may modulate histone function by interfering with cytoplasmic post-transcriptional processes such as acetylation and chaperone binding that are important for proper folding and nuclear transport of histones [30,31].

The interaction of lunasin with histones was associated with alterations in histone acetylation on H4, whereas the acetylation mark interrogated on H3 (H3K9Ac) was not affected by lunasin treatment. With respect to H4 acetylation, lunasin treatment reduced the levels of H4K8Ac and H4K12Ac to similar levels in both H661 and H1299, whereas, the level of H4K16Ac was not affected in H1299 but was significantly higher in H661. These effects on H4K8Ac and H4K12Ac levels are in agreement with studies on lunasin’s effects on the breast cancer cell line MDA-MB-231; however, in contrast to H661, MDA-MB-231cells exhibited a modest 8% decrease in H3K9Ac [19]. Other studies on the effects of lunasin on a immortalized prostate epithelial cells and tumorigenic prostate cancer cells showed H4K8 acetylation was unchanged in both cell lines [21]. However, similar to our results, lunasin did cause an increase in H4K16 acetylation in both the prostate epithelial cell line and the prostate cancer cell line. Interestingly, analysis of the acetylation state H4K16 in the promoter of a pro-apoptotic gene, THBSI, revealed hyperacetylation at this mark in the normal epithelial cells but not in the prostate cancer cells [21]. Based on the specific histone acetylation marks affected by lunasin and direct *in vitro* assays, it is likely that lunasin effects on histone acetylation are due at least in part on inhibiting the activity of the histone acetyltransferases PCAF, p300, and HAT1A [19,21,32]. To date, no functional data are available to clearly demonstrate effects on histone acetylation are important for lunasin effects. However, the specific lunasin-modulated histone acetylation marks have been shown to be important for controlling gene expression and cell proliferation, as well as having potential roles in cancer [33,34,35,36]. One potentially functionally important histone acetylation effect of lunasin is the reduction in H4K12Ac levels. Increased H4K12 acetylation has been associated with poor clinical responses in breast cancer patients [37] and in prostate tumors from patients with metastatic disease [38]. Thus, lunasin’s ability to reduce H4K12Ac levels may reduce the aggressiveness of tumors with the high H4K12Ac phenotype.

Besides effects on histone acetylation, we demonstrated lunasin had cell-line-specific expression patterns for several integrin subunits and distinct interaction profiles with specific integrin subunits that correlated with differential effects on lunasin uptake and integrin signaling. Lunasin interactions with the integrin subunits αv, α5, β1 and β3 were significantly higher in the lunasin-sensitive H661 cells compared to the lunasin-insensitive H1299 cells and were associated with the selective disruption in H661 cells of the interactions of β1 and β3 with pFAK, Kindlin and ILK, key factors required for the initial steps of integrin signaling [39,40]. Immunoblot analyses confirmed lunasin selectively inhibited integrin signaling in H661 cells by reducing levels of pFAK, pAkt, and pERK1/2 compared to controls. These results are consistent with previous studies that demonstrated lunasin interacts with αvβ3 integrin and inhibits Akt activation in human macrophages [15] and that lunasin interacts with α5β1 and inhibits FAK/ERK/NF-κB signaling in colon cancer cells [27]. Given that lunasin disrupted interactions of both β1- and β3-containing integrins with the signaling partners pFAK, ILK and Kindlin in H661 cells, it is possible that lunasin effects in H661 cells involve integrins αvβ3, αvβ1 and α5β1.

Based on the observations that H1299 cells expressed very low levels of the αv subunit; the intensity of lunasin-αv interactions were higher in H661 cells compared to H1299 cells; and both cell lines had similar levels of lunasin-α5β1 interactions, we hypothesized that integrins containing the αv subunit were the major mediators of lunasin’s ability to inhibit proliferation in H661 cells. To test this possibility, we utilized siRNAs specific for the αv subunit in both H661 and H1299 cells. Silencing αv subunit expression in H661 cells caused a dramatic reduction of cell proliferation, demonstrating signaling through αv-containing integrins is important for cell growth in this NSCLC line. However, integrin αv silencing did not completely inhibit cell proliferation, and lunasin was unable to further reduce proliferation in αv-silenced H661 cells. This result in conjunction with the PLA results suggests that in H661, lunasin is antagonizing signaling through αvβ3 and αvβ1. In contrast, silencing the αv subunit in H1299 cells had more modest effects on proliferation, indicating αv-containing integrins are not as important for the *in vitro* proliferation of this cell line. As expected, lunasin did not have an effect in αv-silenced H1299 cells since this line is resistant to lunasin when grown in adherent culture conditions. These results provide the first functional verification that lunasin interactions with integrins are important for its biological activity and confirm αv-containing integrins are a potential target for the development of therapeutics for treating NSCLC. Integrins containing αv have previously been proposed to be important targets for a number of cancer types [41,42] including in glioblastoma [43], prostate cancer [44], melanoma [45], ovarian cancer [46], and lung cancer [47,48]. Integrins are particularly important in mediating cell-matrix interactions and modulating epithelial-mesenchymal transitions during metastasis [49,50,51], thus lunasin may be useful in this context. Initial studies using colon cancer cells indicate this is indeed the case [27].

Our results taken together with previous studies strongly support the notion that lunasin has the ability to inhibit proliferation of cancer cells by serving as an integrin antagonist and by modulating histone acetylation. The effects on integrin signaling appear to involve suppression of phosphorylation-mediated activation of Akt. This effect may serve as a link between inhibition of integrin signaling and histone acetylation given recent studies demonstrating Akt-dependent regulation of histone acetylation in gliomas and prostate cancer [38]. Additional functional studies are required to clearly define the specific lunasin-induced changes in cancer cells responsible for lunasin’s therapeutic effects and to what extent lunasin’s multiple modes of action are linked.

A major open question relating to the use of lunasin as a chemotherapeutic for the treatment of cancer is whether it is sufficiently potent and bioavailable for clinical use. For these studies, we utilized a concentration of 100 µM lunasin, which is relatively high and not likely to be achievable in animals or humans. This was necessary due to the fact that NSCLC cells are significantly more resistant to lunasin treatment when grown in adherent culture conditions compared to non-adherent colony forming assays where the IC_50_ for lunasin is 1.3 µM [14]. Thus, standard *in vitro* assays useful for conducting mechanistic studies such as those in this report underestimate the potential *in vivo* activity of lunasin. This has been borne out in studies using mouse models where lunasin has exhibited significant anticancer effects at doses of 4–30 mg lunasin/kg body weight [11,14,27]. These doses are comparable to those of other biologics used clinically and suggest that lunasin may have therapeutic potential.

## 4. Experimental Section

### 4.1. Cell Lines

Human NSCLC cell lines H661 and H1299 were obtained from the American Type Culture Collection (Rockville, MD, USA). The NSCLC cells were maintained in RPMI 1640-GlutaMAX (Invitrogen, Grand Island, NY, USA) containing 10% FBS, 1 mM sodium pyruvate, 100 IU/mL penicillin and 100 mg/mL streptomycin sulfate at 37 °C in 5% CO_2_.

### 4.2. Reagents

All chemicals were of reagent grade or better and purchased from Sigma-Aldrich (St. Louis, MO, USA). Lunasin was purified by as previously described [4] and was >99% pure.

### 4.3. Lunasin Uptake Immunocytochemistry

H661 and H1299 cells were plated in Nunc™ Lab-Tek™ II 8-well chamber slides (Thermo Scientific, Rockford, IL, USA) at 20,000 cells/cm^2^ in 400 µL medium and incubated overnight at 37 °C in 5% CO_2_ humidified atmosphere. After treatment for 24 h with vehicle (50 mM sodium phosphate, pH 7.4) or 100 µM lunasin, cells were fixed, permeabilized, blocked and stained (1:100 mouse monoclonal anti-lunasin [4] *v*/*v*, 1:100 Alexa Fluor^®^ 488-conjugated AffiniPure goat anti-rabbit *v*/*v*, 1:40 Alexa Fluor^®^ 594-conjugated phalloidin *v*/*v*, 1 µg/mL DAPI using traditional immunocytochemisty techniques. Slides were placed overnight in the dark at 4 °C and analyzed the following day by fluorescent microscopy utilizing the Axio Observer.A1 inverted fluorescent microscope and AxioVision v4.6.3.0 software (ZEISS Microscopy, Thornwood, NY, USA).

### 4.4. Histone Acetylation Analyses

NSCLC H661 and H1299 cells were plated at a density of 5000 cells/cm^2^ in 150 cm^2^ dishes and treated 6 h later with 100 µM lunasin for 48 h. The cells were harvested and homogenized in hypotonic lysis buffer (10 mM Tris-HCl pH 8.0, 1 mM KCl, 1.5 mM MgCl_2_ and 1 mM dithiothreitol) with protease inhibitors (cOmplete Mini Protease Inhibitor Cocktail, 1 tablet/10 mL, Roche, Indianapolis, IN, USA), and histones were extracted using 0.4 N of H_2_SO_4_ as previously described [52]. Protein concentrations were determined using a bicinchoninic acid-based assay, (Pierce™ BCA Protein Assay Kit, Thermo Scientific) using bovine serum albumin (BSA) as a standard. Histone extracts were subjected to SDS-PAGE (10 μg total protein per sample) and immunoblots prepared by electroblotting to polyvinylidene difluoride membranes. Immunoblots were probed with antibodies (EMD Millipore, Billerica, MA, USA) specific for histone H3K9 acetylation (#07-352), H4K8 acetylation (#07-328), H4K12 acetylation (#07-595) and H4K16 (#07-329) using standard methods. Luminescent detection was done using the chemiluminescent substrate SuperSignal^®^ West Femto (Thermo Scientific) and the immunoblots imaged using a Kodak Image Station 4000R Pro equipped with Carestream Molecular Imaging Software v5.0.7.24 (Carestream, Rochester, NY, USA). Histone acetylation signal intensity was quantified by Image J analysis software [53].

### 4.5. In Situ Proximity Ligation Assays (PLA)

H661 and H1299 cells were plated in 8-well chamber slides at 20,000 cells/cm^2^ in 400 µL medium and cultured for 6 h prior to a 24 h treatment with either vehicle or 100 µM lunasin. After treatments, cells were fixed with 4% paraformaldehyde for 10 min and followed by three phosphate buffered saline (PBS, 58 mM Na_2_HPO_4_, 17 mM NaH_2_PO_4_, 6.8 mM NaCl, pH 7.4) washes of 5 min each. Cells were permeabilized with 0.5% Triton X-100 in PBS for 10 min and the cells were washed with 0.05% Tween 20 in TBS three times, 5 min per wash. PLA was performed according to the manufacturer’s protocol using the Duolink Detection Kit (Olink Bioscience, Uppsala, Sweden). For each experiment, duplicate samples were incubated with the appropriate antibody combinations using the following antibodies: lunasin (mouse monoclonal, [4]) combined with the EMD antibodies for histone H3 (#06-755), histone H4 (#07-108), integrin β3 (#AB2984), the Cell Signaling Technology (Danvers, MA, USA) antibodies for integrin α5 (#4705), αv (#4711) or the Abcam (Cambridge, MA, USA) antibody forβ1 (#ab134179); or combinations of Kindlin (#ab68041, Abcam), p-FAK (Tyr397 phosphorylation, #ab4803, Abcam), or ILK (#3862, Cell Signaling Technology) with either integrin β1 (#ab24693, Abcam) or β3 (#ab7167, Abcam). Cells were visualized by fluorescent microscopy using an Axio Observer-A1 inverted fluorescent microscope and AxioVision v4.6.3.0 software (ZEISS Microscopy). All images were collected using identical exposure settings. The background fluorescence was subtracted from the images and the signal intensity was quantified by Image J analysis software [53] and is expressed as fluorescence intensity per cell. A minimum of 20 cells in each duplicate sample were analyzed for each experiment. Three independent experiments were done for each interaction assay.

### 4.6. Integrin Subunit Analysis by Immunoblotting

Both H661 and H1299 cells were grown under conventional anchorage-dependent conditions, harvested and prepared for SDS-PAGE and subsequent immunoblot analysis. Briefly, cells were plated at a density of 6000 cells/cm^2^ in T-75 flasks and incubated for 72 h. Cells were harvested by scraping in ice-cold Dulbecco’s PBS (DPBS), pelleted by centrifugation, and washed once with ice-cold DPBS. Cells were then lysed by resuspending the cell pellet in ice-cold RIPA buffer (50 mM Tris-HCl pH 8.1, 150 mM NaCl, 1% *v*/*v* NP-40, 0.5% *w*/*v* sodium deoxycholate, 0.1% *w*/*v* SDS) supplemented with 1 mM Na_3_VO_4_ and cOmplete Mini Protease Inhibitor Cocktail, and freezing for 1 h at −80 °C. Cell homogenates were then thawed on ice, sonicated for three cycles of 10 s each on ice and the protein concentration determined. Total protein was adjusted to 2 mg/mL in reducing sample loading buffer (62 mM Tris-HCl pH 6.8, 2.5% (*v*/*w*) SDS, 5% (*v*/*v*) β-mercaptoethanol, 10% (*v*/*v*) glycerol, 10 µg/mL bromophenol blue). Samples (20 µg) were then subjected to SDS-PAGE, transferred by electroblotting to polyvinylidene difluoride membranes and probed with the relevant antibodies using standard methods. The primary antibodies used were: Cell Signaling Technology antibodies for integrin α5 antibody (#4705), integrin αv antibody (#4711) and actin (#4970); Abcam antibodies for integrin β1 antibody (#ab52971), integrin β3 antibody (#ab119992), and integrin β5 antibody (#ab15459). Secondary antibodies were from Jackson ImmunoResearch (West Grove, PA, USA): horseradish peroxidase (HRP)-conjugated AffiniPure goat anti-rabbit IgG (#111-035-003), horseradish peroxidase (HRP)-conjugated AffiniPure sheep anti-mouse IgG (#515-035-003), alkaline phosphatase (AP)-conjugated AffiniPure goat anti-rabbit IgG (#111-055-003). Luminescent detection was done using the chemiluminescent substrate SuperSignal^®^ West Femto while colorimetric detection utilized 1-Step™ NBT/BCIP (Thermo Scientific). Immunoblots were imaged using a Kodak Image Station 4000R Pro utilizing Carestream Molecular Imaging Software v5.0.7.24 from Carestream.

### 4.7. Integrin Signaling Immunoblot Analyses

H661 and H1299 were plated at 5000 cells/cm^2^ in 150 cm^2^ dishes and treated with 100 µM lunasin for 24 h. Cells were then washed with PBS, and lysed in RIPA buffer. The protein concentrations of each extract were determined and 40 μg total protein per sample were subjected to SDS-PAGE and immunoblot analysis as described above. Blots were probed with Cell Signaling Technology antibodies for integrin αv (#4711), ILK (#3862), p-Akt (S473 phosphorylation, #4060) Akt (#4691), p-ERK1/2 (Thr202/Tyr204 phosphorylation, #4370), ERK1/2 (#4695), p-GSK3-α/β (Ser21/9 phosphorylation, #9331), and GSK3-α/β (#5676), and Abcam antibodies for p-FAK (Y397 phosphorylation, ab4803), and FAK (abcam, ab40794). Immunoblots were imaged using a Kodak Image Station 4000R Pro equipped with Carestream Molecular Imaging Software v5.0.7.24.

### 4.8. SiRNA-Mediated Knockdown of Integrin αv

Integrin αv siRNAs (#3685) and a scrambled negative control siRNA (#SR30004) were purchased from Origene (Rockville, MD, USA). For transfection with siRNA, cells were plated 5000 cells/cm^2^ in six-well plates and transfected 24 h later using lipofectamin 2000 (Invitrogen) according to the manufacturer’s protocol. At 48 h after transfection, cells were harvested and replated at 5000 cells/cm^2^ prior to treatment with 100 µM lunasin or vehicle (50 mM NaPO_4_ buffer, pH 7.4).

### 4.9. Proliferation Assays

Cell growth was assessed by a tetrazolium-based MTS assay (Cell Titer 96 Aqueous One Solution Assay, Promega, Madison, WI, USA). The cells were seeded in 96-well plates at a density of 5000 cells/cm^2^, and treated with 100 µM lunasin for 24, 48 or 72 h. The MTS assay was performed according to the manufacturer’s instructions. Absorbance readings were made on a Synergy™ H1 hybrid multi-mode microplate reader (BioTek^®^, Winooski, VT, USA).

### 4.10. Statistical Analyses

All studies included at least three independent experiments that included two or three replicates each and the data are expressed as the mean ± SD. Significance differences between treatments were determined using the Student’s t-test and a *p* < 0.05 was used as the criterion for statistical significance. Statistical analysis was performed using SigmaPlot^®^ v11.2 (Systat Software, Inc., Chicago, IL, USA).

## 5. Conclusions

We conclude lunasin inhibits NSCLC cell proliferation by functioning as an integrin signaling antagonist that targets αv-containing integrins; specifically αvβ3 and αvβ1 in H661 cells. Lunasin also has cell-line specific effects on histone acetylation that are correlated with lunasin sensitivity that may also be important for suppressing growth of NSCLC cells. Thus, lunasin exhibits two modes of action that may work in concert to exert lunasin’s therapeutic effects.

## Supplementary Materials

Supplementary figure can be found at http://www.mdpi.com/1422-0067/15/12/23705/s1.
